# Identification of Cytotoxic Flavor Chemicals in Top-Selling Electronic Cigarette Refill Fluids

**DOI:** 10.1038/s41598-019-38978-w

**Published:** 2019-02-26

**Authors:** My Hua, Esther E. Omaiye, Wentai Luo, Kevin J. McWhirter, James F. Pankow, Prue Talbot

**Affiliations:** 1 Environmental Toxicology Graduate Program, University of California, Riverside, Riverside, CA United States; 2 Department of Chemistry and Department of Civil and Environmental Engineering, Portland State University, Portland, Oregon, United States; 3 Department of Molecular, Cell and Systems Biology, University of California, Riverside, Riverside, CA United States

## Abstract

We identified the most popular electronic cigarette (EC) refill fluids using an Internet survey and local and online sales information, quantified their flavor chemicals, and evaluated cytotoxicities of the fluids and flavor chemicals. “Berries/Fruits/Citrus” was the most popular EC refill fluid flavor category. Twenty popular EC refill fluids were purchased from local shops, and the ingredient flavor chemicals were identified and quantified by gas chromatography-mass spectrometry. Total flavor chemical concentrations ranged from 0.6 to 27.9 mg/ml, and in 95% of the fluids, total flavor concentration was greater than nicotine concentration. The 20 most popular refill fluids contained 99 quantifiable flavor chemicals; each refill fluid contained 22 to 47 flavor chemicals, most being esters. Some chemicals were found frequently, and several were present in most products. At a 1% concentration, 80% of the refill fluids were cytotoxic in the MTT assay. Six pure standards of the flavor chemicals found at the highest concentrations in the two most cytotoxic refill fluids were effective in the MTT assay, and ethyl maltol, which was in over 50% of the products, was the most cytotoxic. These data show that the cytotoxicity of some popular refill fluids can be attributed to their high concentrations of flavor chemicals.

## Introduction

Electronic cigarettes (EC) and their refill fluids (also called e-liquids) are relatively new tobacco products. In 2014, consumers could choose from over 400 models of EC and ~8,000 different refill fluid flavor names^1^. While many flavor chemicals in EC are reported safe for use in food^2^, the National Institute for Occupational Safety and Health has warned food-processing workers that some inhaled flavor chemicals may cause lung disease^3^, and the Flavor and Extract Manufacturers Association has strongly cautioned that their “Generally Recognized as Safe” (GRAS) certification is intended for exposure by ingestion, not inhalation^4^.

Information on adverse health effects of ECs comes from several sources. Adverse systemic effects, including inflammatory lung and digestive diseases, have been linked to EC use in case reports^5^. A systematic review on EC health effects collated data on EC flavor chemicals that have cytotoxic effects as well as information on particles, harmful metals, tobacco specific nitrosamines, and toxic carbonyl-containing degradation products^6^. EC users have reported numerous negative effects of vaping on their health^7^. In an *in vitro* study, EC refill fluids varied in their cytotoxicities when tested with embryonic and adult cells; products with high concentrations of flavor chemicals were often the most toxic^8^. Cinnamaldehyde was subsequently identified in the most cytotoxic refill fluids^9,10^ and was found at toxic (*in vitro*) concentrations in a broad spectrum of refill fluids not suggesting “cinnamon”, such as variations of “fruit”, “berry”, “coffee”, “tobacco”, and “sweet”^10^. Cinnamaldehyde was also immunosuppressive when tested with human respiratory cells^11^. Other flavor chemicals in EC refill fluids are also a concern. For example, diacetyl, which can cause *bronchiolitis obliterans*^12,13^, was found in a high percentage of randomly sampled refill fluids with flavor terms related to “buttery”, “caramel”, “fruity”, “alcohol” and “candy”^14,15^. In an air-liquid interface model, toxicity was linked to flavors with “strawberry”-flavored refill fluids being the most cytotoxic^16^.

Existing data suggest that high concentrations of flavor chemicals in EC may harm users. It is important to identify and understand which EC flavor types are commonly purchased, what their chemical compositions are, and what their potential toxicities are. Here we: (1) evaluated EC users’ flavor preferences based on an Internet survey and data from local and Internet vape shops, (2) identified and quantified the flavor chemicals in 20 popular refill fluids, (3) established which of the popular fluids are cytotoxic, and (4) identified the flavor chemical ingredients that are individually cytotoxic at concentrations found in the popular refill fluids.

## Results

### Demographics and flavor preferences of EC users in the online survey

We conducted an online survey to identify the most popular flavors of EC refill fluids. Of 2,753 participants, 853 were current EC users (Supplemental Table 1). Most EC users were between ages 18–22 (49.5%), male (72.0%), and listed “some college” as their highest education (39.0%). The most represented ethnic groups were White/Caucasian (43.0%), followed by Asian (23.4%), and Hispanic/Latino (19.0%). 87.0% were ever cigarette users. 68.0% were ever cigarette users that no longer smoked. 53.1% listed ECs as aids to quit smoking. 49.0% listed nicotine replacement products as aids to quit smoking. EC use was “influenced by friends” (54.0%) and some believed “vaping is safer than smoking” (55.0%) (Supplemental Table 1). Most users described their health as “very good/good” (73.0%) and vaped daily (82.0%) for at least 1 month to 2 years (74.0%). Most participants described their use of EC as “regularly, at least once a day” (57.0%) and for 31–59 minutes a day (22.0%) or 1–3 hours a day (22.0%). In users’ decisions to vape, refill fluid flavors were deemed “very important” or “important” (78.0%). The most popular models of EC were tanks/mods (47.0%). Over half of the EC users (54.0%) currently used EC products, while 19.0% were former users, and 27.0% were dual users of both EC and conventional cigarettes.

**Figure 1:**
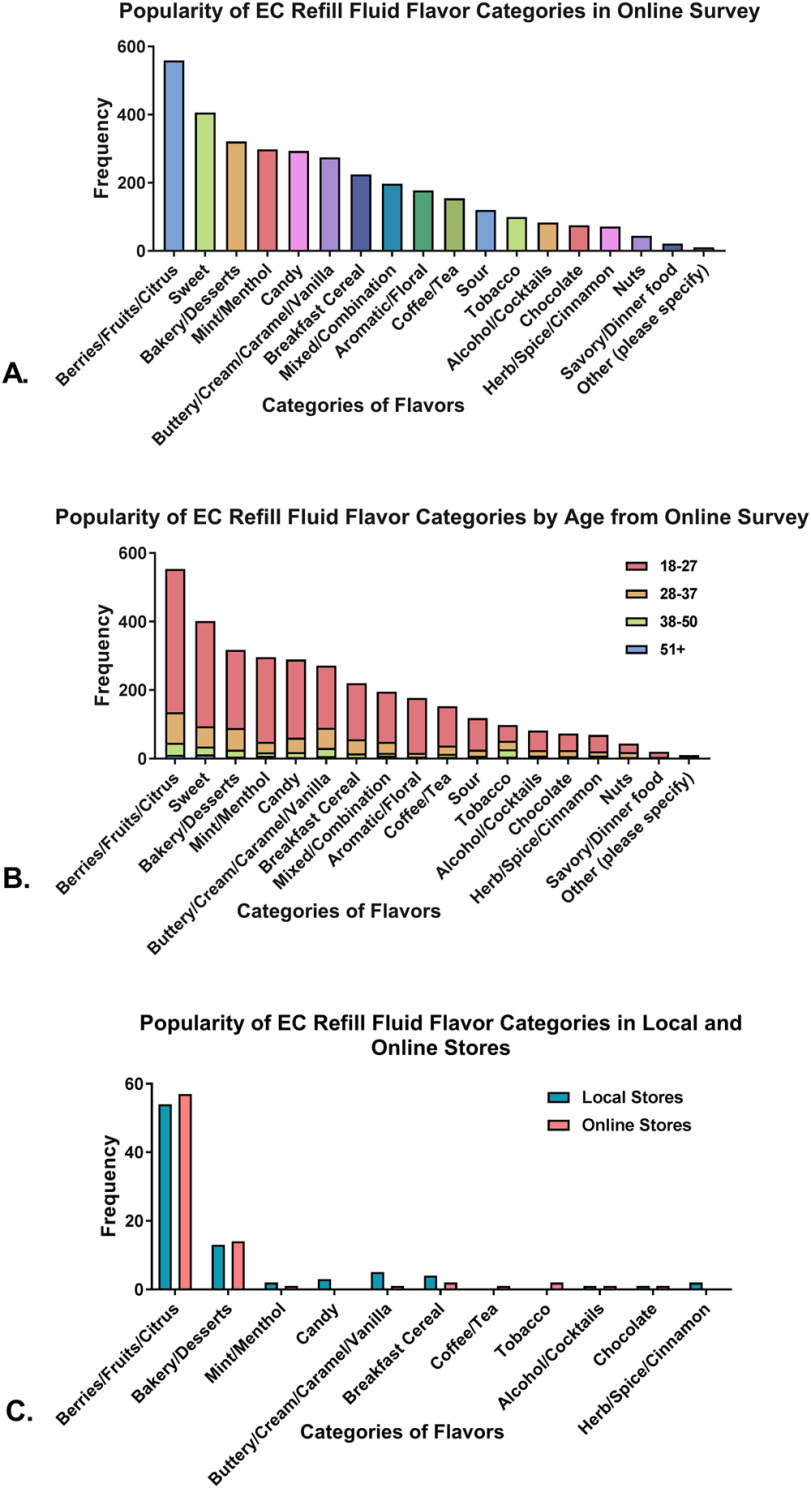
Frequency distribution of popular flavors from survey, local shops, and online stores. (**A**) Results from the online survey. (**B**) Popularity of flavor categories among different age groups in the online survey. (**C**) Results from the local and online stores. Frequency on the *y*-axis refers to the number of times each flavor category (*x*-axis) appeared in the population.

EC users (N = 789) indicated their flavor preferences from 18 possible flavor categories (Fig. 1A). They were able to select more than one flavor. The top six flavor preferences were “Berries/Fruits/Citrus” (N = 559), “Sweet” (N = 406), “Bakery/Dessert” (N = 321), “Mint/Menthol” (N = 298), “Candy” (N = 293), and “Buttery/Cream/Caramel/Vanilla” (N = 274) (Fig. 1A). The two least popular flavors were “Nuts” (N = 44) and “Savory/Dinner Food” (N = 21). Flavor preferences were similar irrespective of the users’ age (Fig. 1B).

### Popular flavors in local and online shops

To confirm the results of the online survey, 17 EC vape shops in southern California were contacted by telephone or visited to obtain information on their top-selling refill fluids. Each local shop reported 5–10 top-selling refill fluid categories, the majority of which were “Berries/Fruits/Citrus” (54 local; 57 online) (Fig. 1C). The Internet was used to determine flavor profiles when shops could not provide these data. In addition, nine popular online shops were visited, and the flavor profiles for 5–10 top-selling fluids were identified as “Berries/Fruits/Citrus” (Fig. 1C). Flavor categories that were not among the most popular are not included in Fig. 1C.

Based on the above data, 20 top-selling refill fluids were purchased from four shops in Riverside County, CA. One local shop specialized in “cloning” brand-name EC fluids, while the other shops sold products that were made by refill fluid manufacturers. We distinguish these products as “cloned” and “authentic”, respectively. It is important to consider cloned products because they are often less expensive than their authentic branded counterparts, and some shops sell mainly cloned products. Supplemental Table 2 shows the flavor profile and general flavor category for each product purchased in local shops.

### Identification and quantification of flavor chemicals in the 20 popular refill fluids

**Figure 2:**
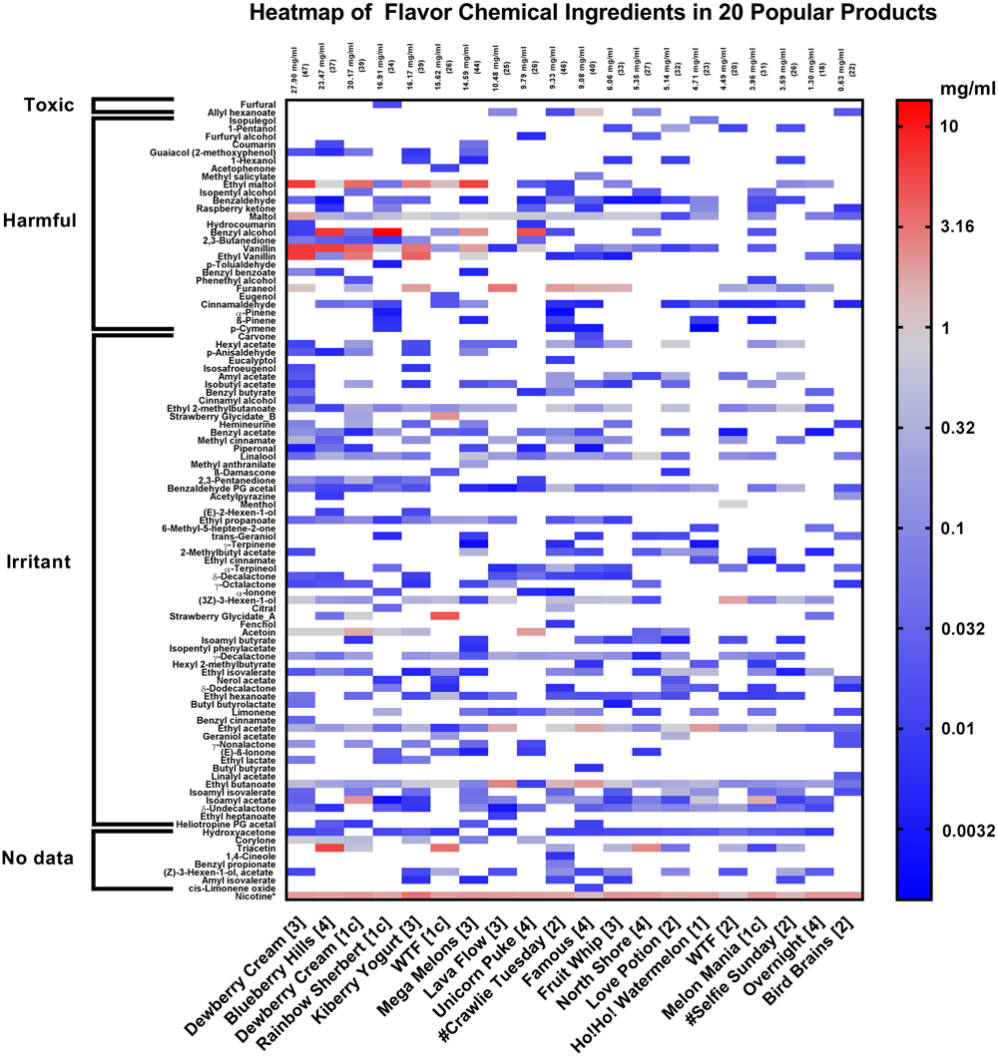
Heat map showing flavor chemical concentrations in 20 popular refill fluids. Chemicals are ordered on the *y*-axis according to their toxicity (based on LD_50_ data from oral exposure in rats) and within each class, they are ranked from most to least toxic. Products (*x*-axis) are ordered according to the weight (mg/ml) of all the flavor chemicals in each product with the highest concentration at the left. Numbers 1–4 with product names denote stores where refill fluids were purchased, and “C” indicates a cloned product. “Rainbow Sherbet” is a clone of “Unicorn Puke” and “Melon Mania” is a clone of “Mega Melons”. The total chemical concentration (mg/ml) and the number of individual chemicals is indicated at the top of each column. Nicotine, which is not a flavoring, is in the bottom row for comparison.

A total of 99 flavor chemicals were identified and quantified in the 20 EC refill fluids purchased in local shops (Fig. 2; Supplemental Table 3). The general flavors associated with each chemical are given in Supplemental Table 3, and the target flavors not found in any of the products are given in Supplemental Table 4. The total concentration of the flavor chemicals in each product, which is given at the top of the columns in the heat map (Fig. 2), ranged from 0.63 mg/ml (“Bird Brains”) to 27.9 mg/ml (authentic “Dewberry Cream”). The *x*-axis of the heat map is sorted based on the total flavor chemical concentration (highest on the left).

On the *y*-axis of the heat map, the 99 chemicals were ranked by their safety classification (Toxic, Harmful, Irritant, and No data) as posted on the Good Scents Flavor Company website^17^, which provides peer-reviewed information for the flavor, food, and fragrance industry. Within each safety classification, chemicals are listed from most to least toxic based on rat oral LD_50_, also posted on the Good Scents website. For most flavor chemicals, one LD_50_ value was available, but if multiple were given, we chose the LD_50_ value reported in the journal of *Food and Cosmetics Toxicology*. Rat oral data were used for ranking because they were available for most chemicals in the heat map, while inhalation LD_50_ data were seldom available. The *y*-axis ranking was useful for predicting which chemicals would be most toxic and therefore most interesting to pursue; however, it is not intended to imply that the chemicals in refill fluids produce the same effects as in the rat oral data. The chemicals with the highest concentrations and highest predicted toxicities are in the upper left quadrant of the heat map.

“Bird Brains” had the fewest flavor chemicals (N = 22), while authentic “Dewberry Cream” had the most (N = 47). In some cases, these chemicals were very low in concentration (e.g., maltol in “Bird Brains”), while in others the concentrations exceeded 1 mg/ml (e.g., ethyl maltol in “Dewberry Cream”). Thirteen percent of the flavor chemicals were present at concentrations higher than nicotine in some samples.

**Figure 3:**
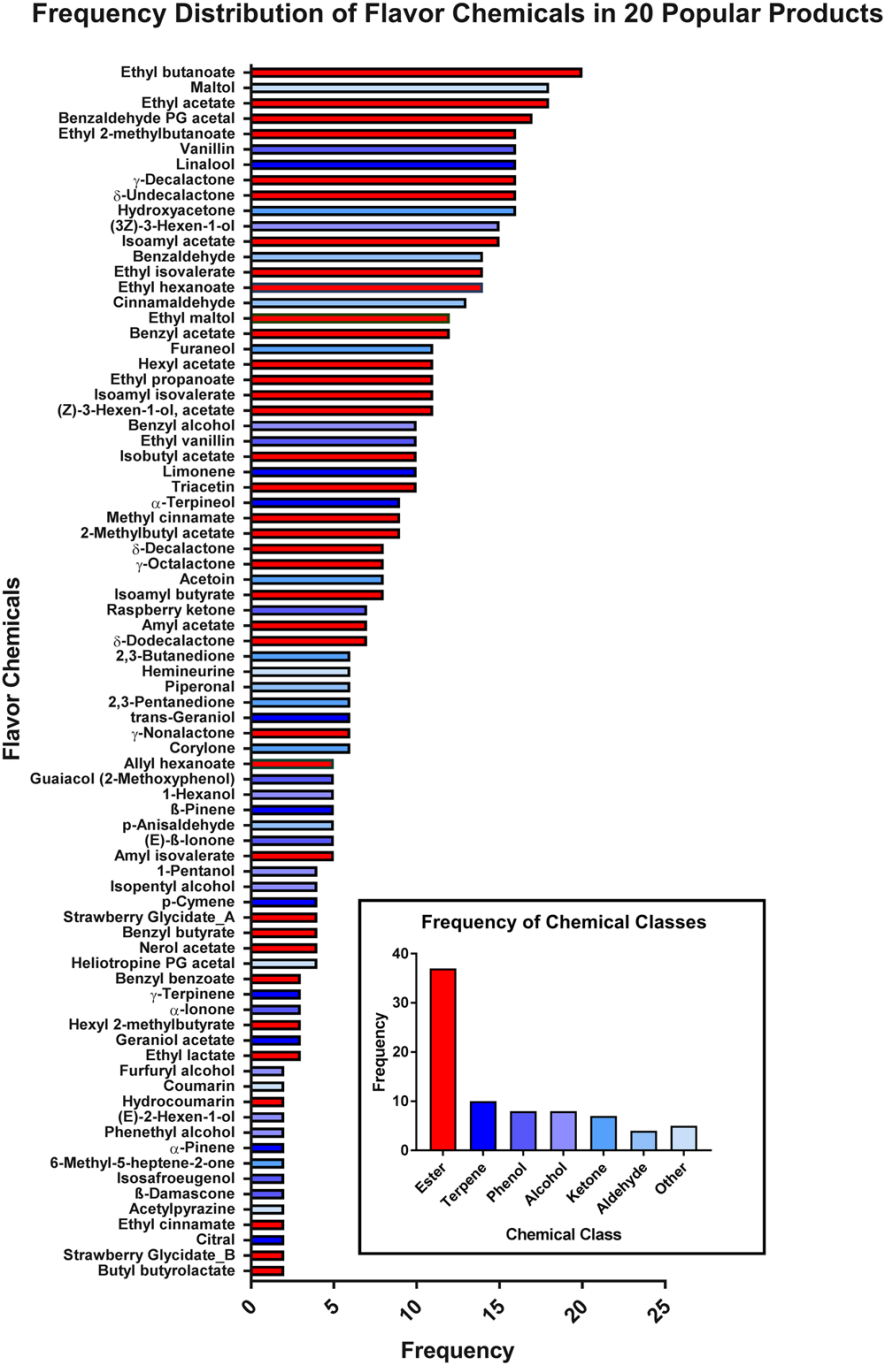
Frequency distribution of flavor chemicals within popular products and their chemical class. Chemicals are ranked according to their frequency in popular products for all data. The inset shows the class to which each chemical belongs.

The frequency with which individual chemicals were found in the 20 popular products varied. Some were found in all or almost all refill fluids (e.g., maltol and ethyl acetate), while others were only in 2–3 products (e.g. ethyl lactate and citral) (Fig. 3). Of the 99 chemicals identified in the popular products, 28 appeared in at least 10 of 20 products, indicating that a subset of flavor chemicals is used frequently. Those chemicals that appeared in only one product are shown in Supplemental Table 5.

Data were also analyzed according to their chemical class (Fig. 3 insert). Most flavor chemicals were esters, and many were terpenes, phenols, alcohols, ketones, and aldehydes. The “other” category included benzopyrone, pyrazine, pyrone, and thiazole. While not shown in Fig. 3, some chemicals belong to more than one class, such as vanillin, which is both an aldehyde and phenol.

**Figure 4:**
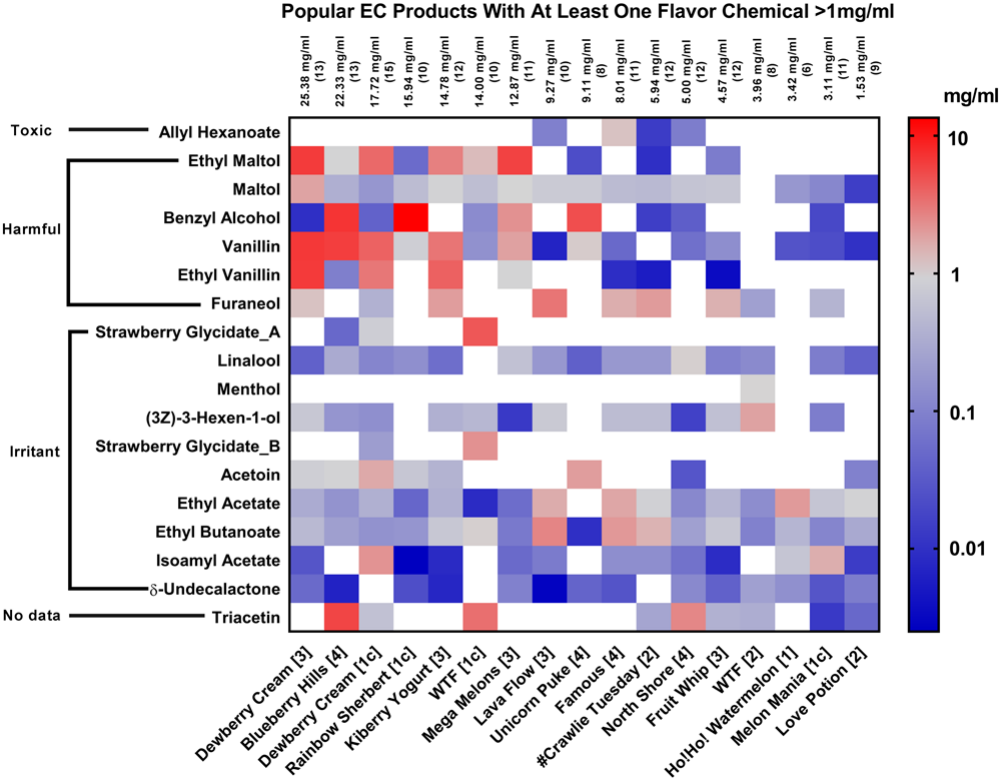
Heat map of popular EC refill fluids with at least one flavor chemical ≥1 mg/ml. These flavor chemicals were considered to be dominant in the popular refill fluids that were analyzed. They are ranked on the *y-*axis according to rat oral toxicity and on the *x-*axis according to total concentration (mg/ml) of the flavor chemicals.

In Fig. 4, the flavor chemical data were filtered to include only those refill fluids (17 of 20) that had at least one chemical at a concentration ≥1 mg/ml. Filtering at this level reduced the number of flavor chemicals from 99 to 18, which we further considered in this study.

### Identification of cytotoxic EC refill fluids

**Figure 5:**
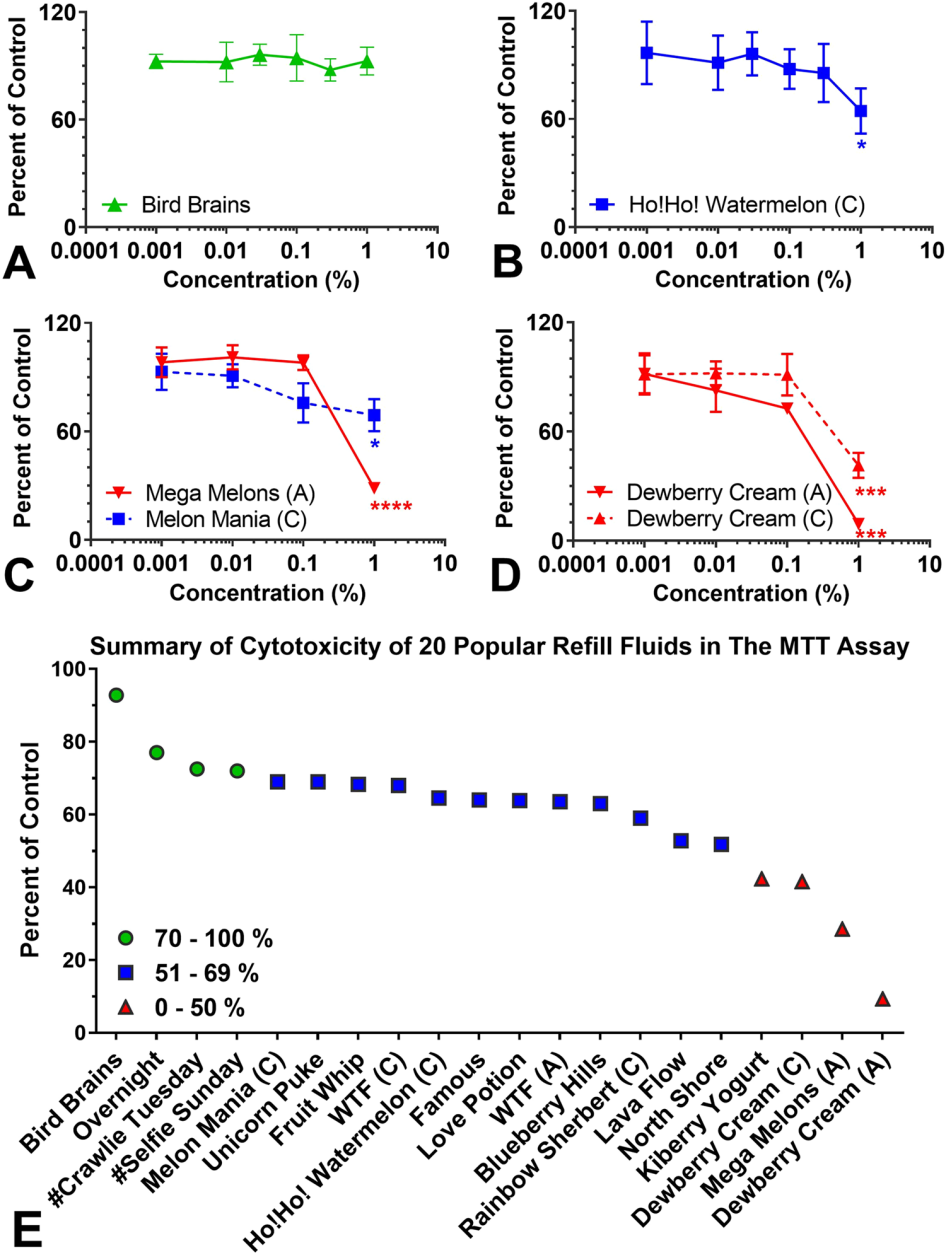
Cytotoxic refill fluids identified using mNSC. (**A**–**D**) Representative MTT concentration- response curves for products that were: (**A**) not cytotoxic, (**B**) cytotoxic reaching IC_70_, and (**C,D**), highly cytotoxic reaching IC_50_. Summary of cytotoxicity screening results showing products that had little effect (green dots), reached an IC_70_ (blue squares), or reached an IC_50_ (red triangles). The most cytotoxic products were “Dewberry Cream”, “Dewberry Cream” clone, “Mega Melons”, and “Kiberry Yogurt”. “Rainbow Sherbet” is a clone of “Unicorn Puke” and “Melon Mania” is a clone of “Mega Melons”. Each graph is the mean ± the standard error of the mean for three independent experiments. *p < 0.05, ***p < 0.001, ****p < 0.0001.

The cytotoxicities of the 20 popular refill fluids were evaluated using the MTT assay, which measures mitochondrial reductase activity. Decreases in the MTT assay relative to untreated controls are indicative of cytotoxicity due to decreases in mitochondrial metabolism and/or cell survival. The concentrations required for a 30% (IC_70_) and 50% (IC_50_) reduction in the MTT assay were determined for each refill fluid (Fig. 5; Supplemental Fig. 1). Some products (e.g., “Bird Brains”) showed no cytotoxicity (Fig. 5A; Supplemental Fig. 1A). Most refill fluids (e.g., “Ho!Ho! Watermelon”) reached at least an IC_70_ (30% inhibition vs control), indicating they were cytotoxic by ISO standard 10993-5^18^ (Fig. 5B). Four refill fluids (“Dewberry Cream”, “Dewberry Cream” clone, “Mega Melons”, and “Kiberry Yogurt”) (Fig. 5C–E) reached at least IC_50_ values (50% inhibition vs. control), again indicating cytotoxicity. Figure 5E summarizes the cytotoxicity data relative to the untreated control for cells treated with a 1% concentration of each refill fluid. Table 1 shows the IC_70_ and IC_50_ values for all 20 products. When tested independently, propylene glycol, glycerol, and nicotine were not cytotoxic at concentrations found in the 1% refill fluid solutions (Supplemental Fig. 2).

**Table 1:** Inhibitory concentrations (IC_70_ and IC_50_) of EC Refill Fluids.

Code	Popular Fluids	mNSC	
		IC_70_^a^ (mg/ml)	IC_50_^b^ (mg/ml)
**A**	“Dewberry Cream (A)”	0.13	0.18
**B**	“Mega Melons”	0.42	0.59
**C**	“Dewberry Cream (C)”	0.46	0.72
**D**	“Kiberry Yoghurt”	0.43	0.71
*E*	“North Shore”	0.36	>1
*F*	“Lava Flow”	0.55	>1
*G*	“Unicorn Puke (C)”	0.37	>1
*H*	“Blueberry Hills”	0.61	>1
*I*	“WTF (A)”	0.64	>1
*J*	“Love Potion”	0.64	>1
*K*	“Famous”	0.57	>1
*L*	“Ho!Ho! Watermelon (C)”	0.68	>1
*M*	“WTF (C)”	0.96	>1
*N*	“Fruit Whip”	0.90	>1
*O*	“Unicorn Puke (A)”	0.97	>1
*P*	“Melon Mania (C)”	0.84	>1
Q	“#Selfie Sunday”	>1	>1
R	“#Crawlie Tuesday”	>1	>1
S	“Overnight”	>1	>1
T	“Bird Brains”	>1	>1

^a^Code alphabet colors matches summary of cytotoxicity of popular refill fluids in the MTT assay (Fig. 5E). Bold = 0–50%, Italic = 51–69%, and Underline = 70–100%.

^b^IC_70_ values read directly off the dose response curves.

^c^IC_50_ values obtained after non-linear fit using log (inhibitor) vs. normalized response - variable slope model.

### Relationship between cytotoxicity and the total number and total concentration of flavor chemicals

**Figure 6:**
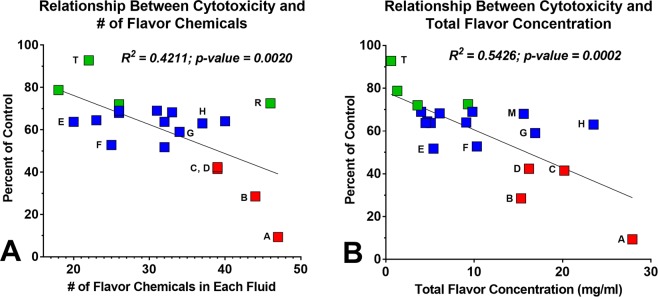
The relationship between cytotoxicity and the total number of flavor chemicals and the total concentration of flavor chemicals. Cytotoxicity is plotted as a function of the total number of flavor chemicals (**A**) and the total concentration of flavor chemicals (**B**) in each of the popular refill fluids. Green dots indicate refill fluids that were not significantly cytotoxic, blue dots are refill fluids that reach an IC_70,_ and red dots are refill fluids that reached an IC_50_. Letters with each point correspond to the products listed in Figs 8 and 9.

Cytoxicity was examined as a function of the total number of flavor chemicals (Fig. 6A) and total concentration of flavor chemicals (Fig. 6B) in each product. The correlations (R^2^) between cytotoxicity and the total number of flavor chemicals in a refill fluid or the total concentration of flavor chemicals in each product were 0.42 and 0.54, respectively. The p values of the correlation coefficients were 0.002 (Fig. 6A) and 0.0002 (Fig. 6B), indicating they were statistically significant.

### Identification of cytotoxic flavor chemicals

**Figure 7:**
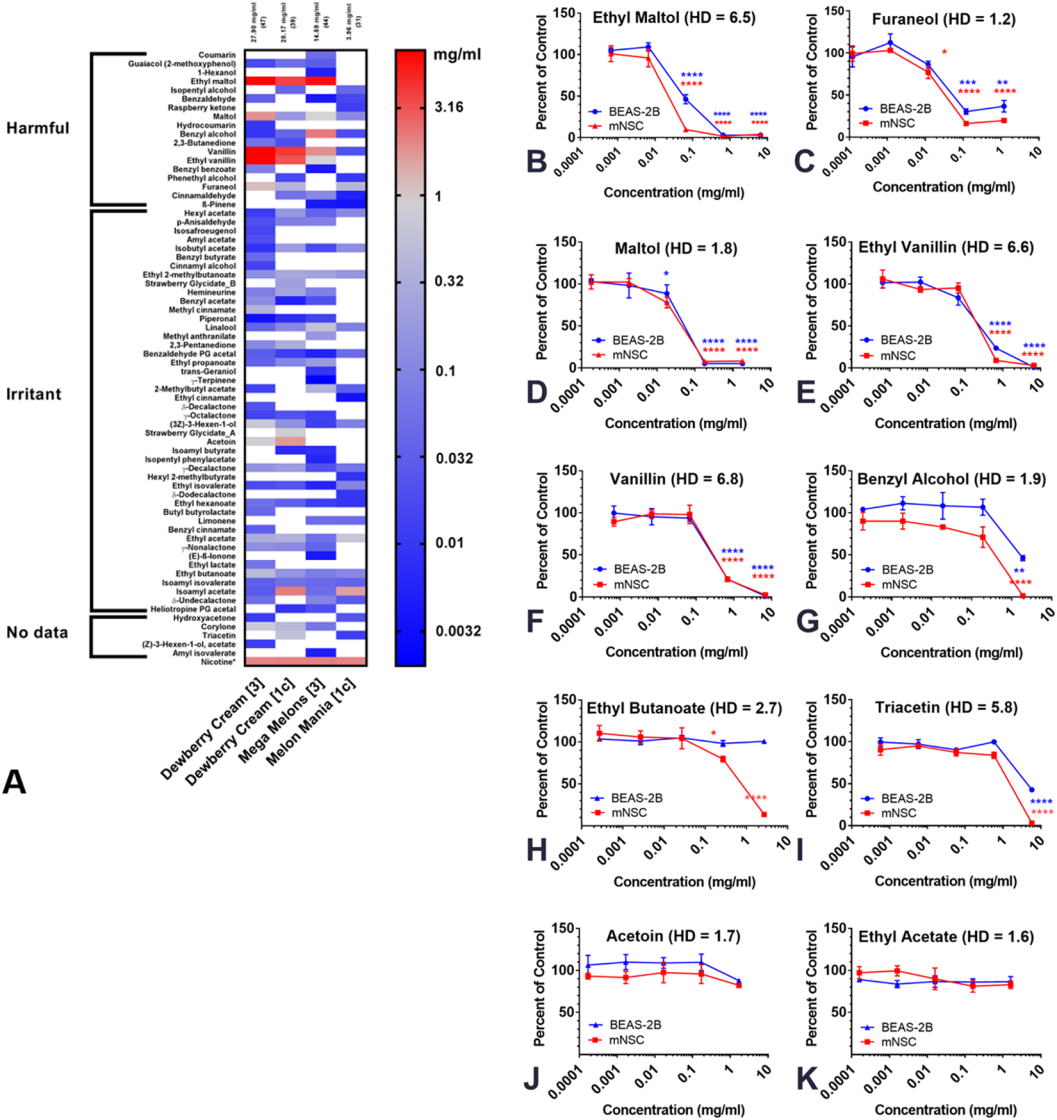
Chemicals in “Dewberry Cream” and “Mega Melons” and their cytotoxicity. (**A**) Heat map showing the flavor chemicals and their concentrations in the two most cytotoxic refill fluids and their clones. Chemicals are ordered on the *y*-axis according to their toxicity and within each class, they are ranked from most to least toxic. Products (*x*-axis) are ranked according to the total flavor chemical concentration, with the highest on the left. The total flavor chemical concentration and number of individual flavor chemicals are indicated at the top of the heat map. Nicotine is in the bottom row for comparison. (**B**–**G**) Concentration-response curves of authentic standard chemicals present in the highest concentrations in the two most toxic refill fluids and their clones. (**H–K**) Concentration-response curves for four chemicals frequently used or present at over 1 mg/ml in refill fluids. Each graph is the mean ± the standard error of the mean for three independent experiments. *p < 0.05, **p < 0.01, ***p < 0.001, ****p < 0.0001. HD = high dose tested.

Figure 7A allows a direct comparison of the two most cytotoxic authentic refill fluids and their corresponding clones. Total flavor concentrations in “Dewberry Cream” (27.9 mg/ml) and its clone (20.17 mg/ml) were similar; however, “Mega Melons” (authentic) had a higher total flavor concentration (14.59 mg/ml) than its clone (“Melon Mania”) (3.96 mg/ml). In no case were the flavor chemicals in the clones an exact match in number or concentration to their authentic counterpart.

We hypothesized that chemicals that were high in concentration in the upper region of Fig. 7A would contribute to the cytoxicity observed in the MTT assay. Six chemicals (ethyl maltol, maltol, vanillin, ethyl vanillin, benzyl alcohol, and furaneol) were >1 mg/ml in “Dewberry Cream” and/or “Mega Melon” and slightly lower in the less toxic clones. Authentic standards of these chemicals were tested in the MTT assay using mNSC and human BEAS-2B cells (Fig. 7B–G). The highest concentration for each authentic standard was chosen to match the highest concentration found in authentic “Dewberry Cream” and “Mega Melons”. In support of our hypothesis, all six authentic standards were cytotoxic at the concentrations found in the refill fluids. mNSC were slightly more sensitive than BEAS-2B to ethyl maltol and benzyl alcohol. For all other chemicals, concentration-response curves were similar for the two cell types. Based on the IC_50_ data, the chemicals that were the most toxic from high to low were: ethyl maltol, furaneol, maltol, ethyl vanillin, vanillin, and benzyl alcohol.

Although their predicted toxicities based on the rat-oral data were lower than the chemicals in the above assays, ethyl butanoate, triacetin, acetoin, and ethyl acetate, were evaluated in a secondary MTT screen (Fig. 7H–K). Both ethyl butanoate and triacetin were cytotoxic at the highest concentrations found in the 20 products, while the other two chemicals were not cytotoxic.

**Figure 8:**
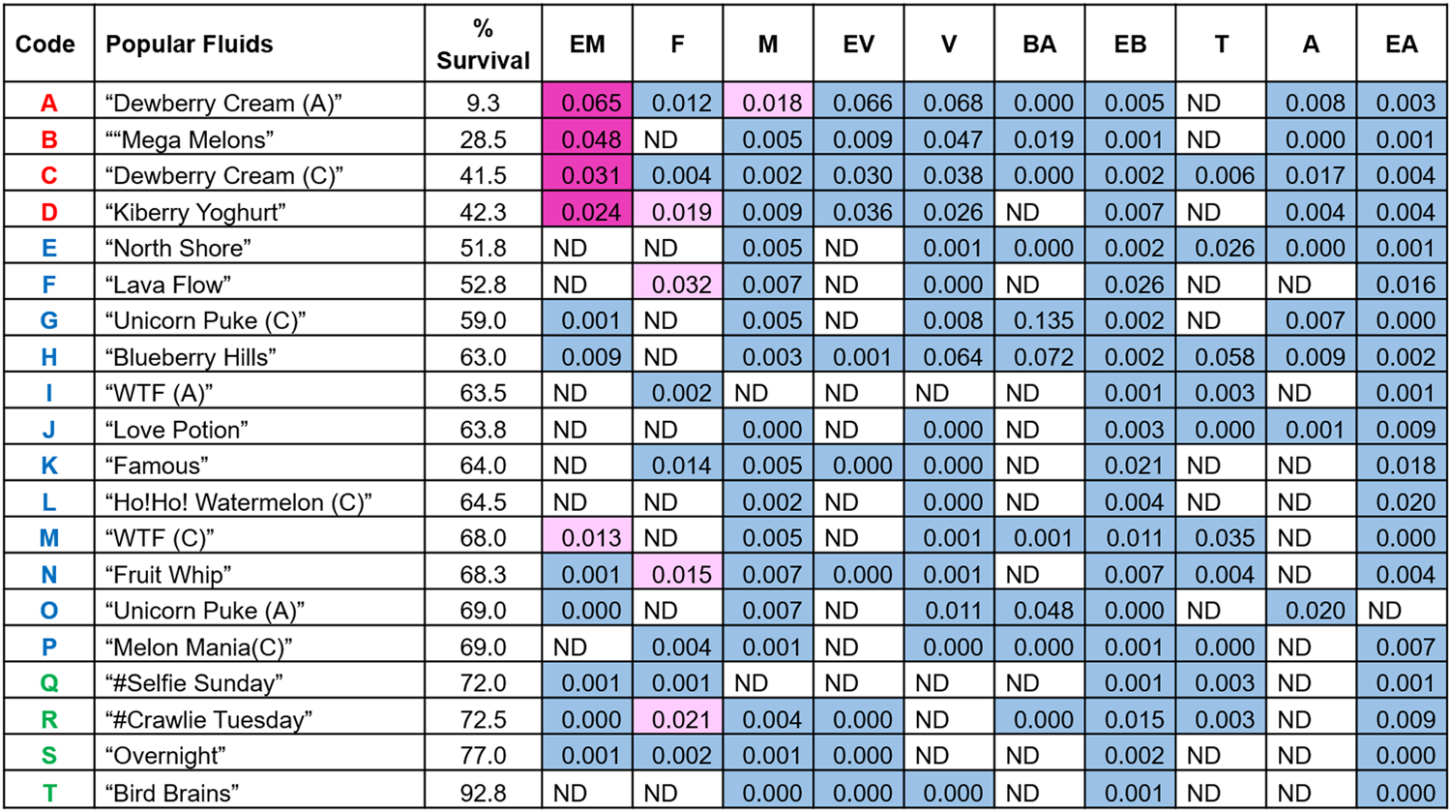
Concentrations (mg/ml) of flavor chemicals in 1% refill fluids and their cytotoxicity. Color code indicates the cytotoxicity of flavor chemicals at the concentrations found in 1% refill fluids. Magenta = concentrations that would reach an IC_50_; Light pink = concentrations that would reach an IC_70_, Blue = no cytotoxic effect. ND = indicates chemical was not detected in the GC-MS analysis. Code alphabet colors match summary of cytotoxicity of popular refill fluids in the MTT assay (Fig. 5E). Red = 0–50%, Blue = 51–69%, and Green = 70–100%. Flavor names: EM = ethyl maltol; F = furaneol; M = maltol; EV = ethyl vanillin; V = vanillin; BA = benzyl alcohol; EB = ethyl butanoate; T = triacetin; A = acetoin; EA = ethyl acetate.

Figure 8 shows the cytotoxicity for each of the refill fluids at 1%, the concentration of each flavor chemical at 1%, and the cytotoxicity of each flavor chemical based on the authentic standard data. In general, when the parent refill fluid was cytotoxic at 1%, it contained flavor chemicals that could account for its cytotoxicity (e.g., “Dewberry Cream” had a cytotoxic level of ethyl maltol). Exceptions to this, such as “North Shore”, which was cytotoxic (51% of control) but did not have a cytotoxic level of flavor chemicals, suggest that our target list of chemicals does not contain some of the flavor chemicals that are used in refill fluids, that chemicals act additively or synergistically to produce cytotoxicity, or that another factor, such as a metals^19^, caused cytotoxicity in this product.

**Figure 9:**
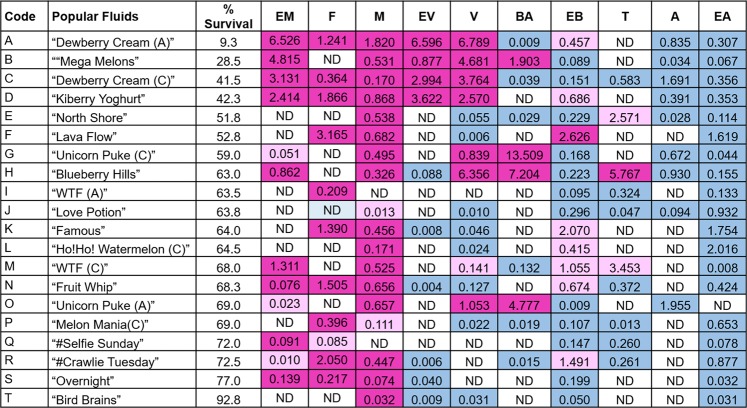
Projected cytotoxicity of flavor chemicals at concentrations (mg/ml) found in refill fluids. Color code indicates the projected cytotoxicity of flavor chemicals at the concentrations found in refill fluids. Magenta = concentrations that would reach an IC_50_; Light pink = concentrations that would reach an IC_70_, Blue = no cytotoxic effect. ND = indicates chemical was not detected in the GC-MS analysis. Flavor names: EM = ethyl maltol; F = furaneol; M = maltol; EV = ethyl vanillin; V = vanillin; BA = benzyl alcohol; EB = ethyl butanoate; T = triacetin; A = acetoin; EA = ethyl acetate.

Since refill fluids are used in ECs without dilution (100%), Fig. 9 is included to show the actual concentration of each flavor chemical in the undiluted parent refill fluid and the cytotoxicity that would be predicted for each chemical at the actual concentration used by EC vapers to produce aerosol. At actual flavor concentrations, all refill fluids would be predicted to be cytotoxic. Ethyl maltol, furaneol, and maltol were always present at concentrations that would be cytotoxic, and these three chemicals were used frequently (maltol for example was in 18 of 20 products tested).

### Relationship between the cytotoxicity of each refill fluid at a 1% concentration and each authentic standard chemical

Each highly cytotoxic refill fluid contained one or more of the six toxic flavor chemicals at concentrations that were as cytotoxic as authentic standards in the MTT assay (Fig. 8). In general, moderately cytotoxic refill fluids had lower concentrations (e.g., “Lava Flow” and “WTF” clone) or non-cytotoxic concentrations (e.g., “Blueberry Hills” and “Unicorn Puke”) of the six toxic chemicals. Non-cytotoxic products or those that did not differ from the control by more than 30% either had none of the six toxic flavor chemicals (e.g., “Bird Brains”) or had low levels (e.g., “Overnight”). These data demonstrate a positive relationship between the concentration of ethyl maltol and the cytotoxicity of the refill fluids in which it was used.

**Figure 10:**
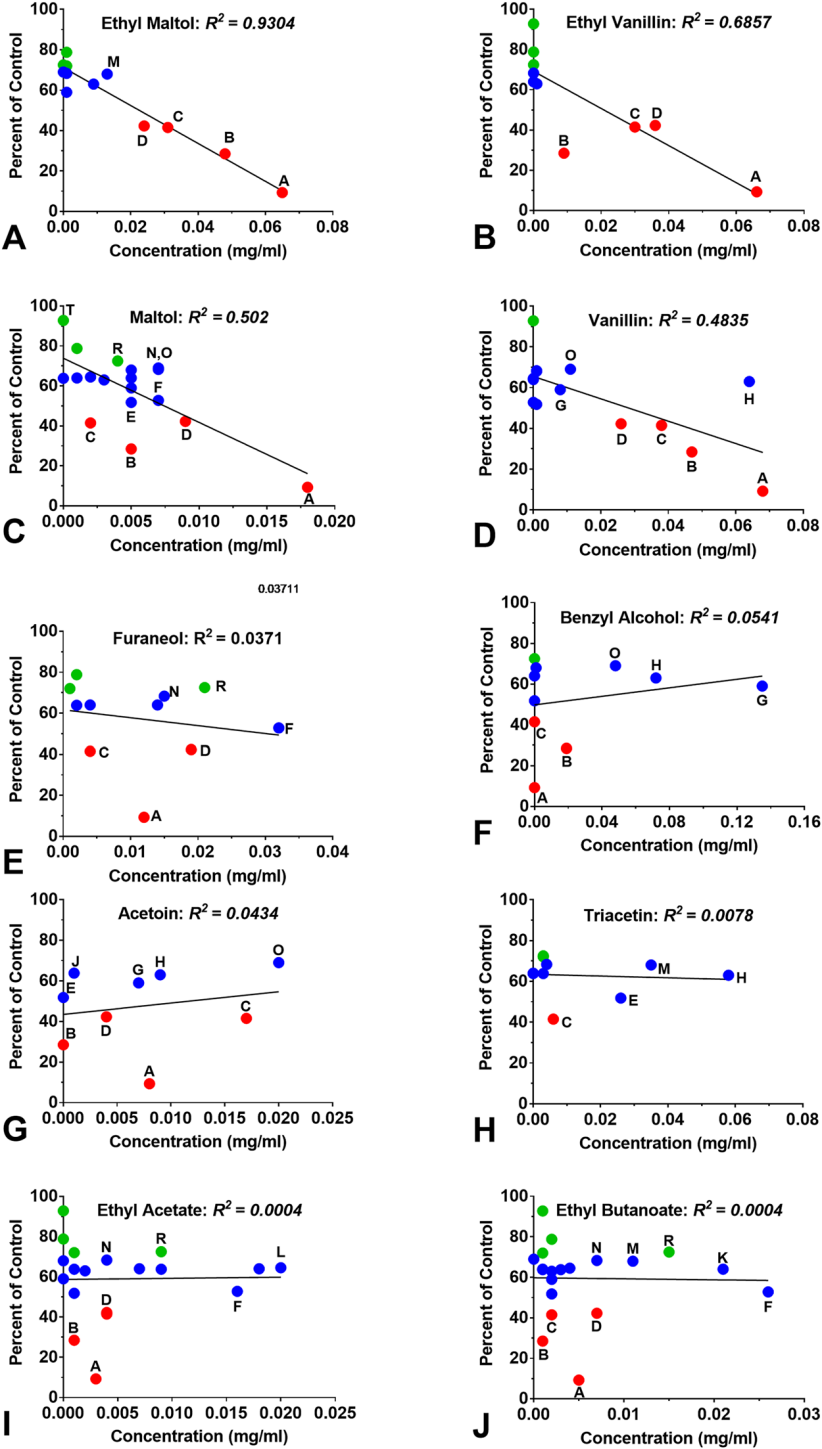
Relationship between the cytotoxicity of each refill fluid at 1% concentration and the concentrations of each authentic standard chemical. Green dots indicate refill fluids that were not significantly cytotoxic, blue dots are refill fluids that reach an IC_70,_ and red dots are refill fluids that reached an IC_50_. Letters associated with dots correspond to products in Fig. 8. Because refill fluids are mixtures of cytotoxic chemicals, only ethyl maltol (the most toxic of the authentic standards) had a high correlation coefficient. The p values for ethyl maltol, maltol, ethyl vanillin and vanillin indicate that the correlations are statistically significant. Correlation coefficients for the other chemicals were affected by the presence of ethyl maltol.

The cytotoxicity of refill fluids (1% concentration) was plotted as a function of the flavor chemical concentration in each fluid at 1% (Fig. 10A–J). Dots are color-coded to toxicity of the refill fluids (red = highly cytotoxic, blue = moderately cytotoxic, green = non-cytotoxic), and the letter code with each dot correlates to a refill fluid in Fig. 8. The cytotoxicity and ethyl maltol concentrations in each fluid were highly correlated (R^2^ = 0.93; p value =  < 0.0001). This high correlation occurs because ethyl maltol was the most cytotoxic of the chemicals tested and it maintained its toxicity when tested in a refill fluid. The correlation coefficient was also significant for ethyl vanillin (R^2^ = 0.68; p value = 0.0033), maltol (R^2^ = 0.502; p value = 0.0010) and vanillin (R^2^ = 0.49; p value = 0.0028), but decreased and was not significant for the remainder of the toxic chemicals (Fig. 10E–J).

## Discussion

The online survey and identification of the top-selling refill fluids in local and Internet shops showed that EC users prefer “Berries/Fruit/Citrus” flavors with “Sweet”, “Candy”, “Bakery/Dessert”, and “Breakfast Cereal” also being popular. In the local and online shops, all top-selling products were in the “Berries/Fruit/Citrus” category. This is the first time that flavor popularity has been assessed using three independent methods, which proved to agree with each other and with other flavor surveys^20–22^. Identification of the popular flavor categories enables research to focus on those that are most relevant to EC users. Our study also identified popular EC products that are cytotoxic, examined the flavor chemicals in the most popular selling refill fluids, and identified specific flavor chemicals that contribute to cytotoxicity at concentrations found in the refill fluids. The dominant flavor chemicals (those ≥1 mg/ml) that we identified in popular refill fluids are important because some, such as ethyl maltol and vanillin, were used frequently at cytotoxic concentrations. Ethyl butanoate, an inexpensive fruity flavoring widely used in the food industry, was in all 20 products, in some cases at cytotoxic concentrations.

The total flavor chemical concentrations exceeded 1 mg/ml (about 0.1%) in all top-selling refill fluids, except in “Bird Brains” (0.63 mg/ml). It is possible that “Bird Brains” had additional flavor chemicals that were not on our target list, and hence not quantified. Also noteworthy, total flavor chemical concentrations exceeded that of nicotine (1.2–3.3 mg/ml) in 19 of 20 products. These data identify flavor chemicals as major constituents of popular refill fluids and further show that the concentrations of flavor chemicals used by manufacturers vary significantly among products.

The total number of flavor chemicals identified (N = 99) in the 20 popular refill fluids is relatively small considering that there are thousands from which manufacturers could choose^2^. These data suggest that EC refill fluid manufacturers use a small subset of the available flavor chemicals in their products, which should help focus future research in this area. The majority of the flavor chemicals were esters, a class that imparts fruity flavors and aromas^23^, consistent with fruity and berry flavors being the most popular. Those flavor chemicals that were <1 mg/ml were likely introduced as minor constituents or impurities in the flavor chemical ingredients used to compound the refill fluids or as accents in a more complex flavoring base.

Our study examined the toxicity of refill fluids and authentic standards of the pure flavor chemicals at concentrations similar to those found in refill fluids. 12 of 20 (60%) popular refill fluids produced an IC_70_ and four produced an IC_50_ in the MTT assay (Table 1). These 16 products (80% of total tested) would be classified as cytotoxic by ISO protocol #10993-5^18^. While some earlier studies reported little cytotoxicity for refill fluids and their aerosols^24,25^, in our study and other recent papers, refill fluids frequently produced cytotoxicity when tested *in vitro*^8–10,16,26^. Our study further showed that the cytotoxicity of the refill fluids was correlated with the total number and the total concentration of flavor chemicals.

The cytotoxicity observed in most refill fluids was isolated to individual flavor chemicals that were tested as authentic standards. The overall hierarchy of potency for the 10 tested chemicals was: ethyl maltol > furaneol > maltol > ethyl vanillin > vanillin > benzyl alcohol > ethyl butanoate > triacetin > acetoin > ethyl acetate. The toxicity of refill fluids was correlated with the concentration of ethyl maltol, ethyl vanillin, maltol and vanillin, further supporting the idea that toxicity, as measured in the MTT assay, was due to the flavor chemicals. The low correlation coefficient of the remaining six chemicals (e.g., benzyl alcohol) and refill fluid toxicity was due to the presence of the more cytotoxic flavor chemicals, such as ethyl maltol. As an example, the products that had benzyl alcohol also had cytotoxic concentrations of ethyl maltol and maltol that reduced R^2^ (Fig. 10F).

As a further example of the correlation between flavor chemicals and cytotoxicity, “#Crawlie Tuesday” had a high number and high concentration of flavor chemicals but had only one flavor chemical (furaneol) that would cause cytotoxicity at a 1% concentration (Fig. 8). Interestingly, the predicted cytotoxicity of #Crawlie Tuesday at 1% test solution based on its furaneol concentration would be ~IC_70,_ and the actual measured inhibitory concentration for this product was 72.5%. The predicted and observed values are remarkably close further supporting the idea that toxicity can be attributed to furaneol.

While heating refill fluids can increase toxicity by formation of carbonyls through decomposition of flavor chemicals or glycerol/propylene glycol^27,28^, the authentic flavor chemicals examined in our study showed toxicity independent of reaction products produced by heat. Maltol and ethyl maltol are especially important as they were detected in >50% of our refill fluids, and they were among the most toxic of the authentic standards tested. Other studies have also reported that vanillin, ethyl vanillin, and ethyl maltol are often used in EC products^28,29^, further supporting the idea that refill fluid manufacturers use a relatively small subset of the flavor chemicals available. Potential harm due to flavor chemicals is further supported by an *in vitro* study in which maltol increased secretion of IL-8 from BEAS-2B cells and decreased barrier function in human bronchial epithelial cells^30^ and by animal studies in which maltol produced long-term adverse health effects in rats and dogs^31^ and elicited liver and kidney damage in mice^32^.

While we have focused on flavor chemicals present in refill fluids at high concentrations, some flavor chemicals may be harmful at low doses. 2,3-Butanedione (diacetyl) was present in 6 of 20 products at concentrations of 0.0187–0.0989 mg/ml, and the related flavorings, acetoin and 3,2-pentanedione, were in 8 of 20 and 6 of 20 refill fluids, respectively. Others have reported 2,3-butanedione in refill fluids^15^ and in EC aerosols^14^, also at relatively low concentrations. Although these chemicals were generally minor constituents (<1 mg/ml), 2,3-butanedione is of concern because it has been linked to *bronchiolitis obliterans* (popcorn lung)^12,13^.

Our data do not address the toxicity of flavor chemicals in aerosols. However, we have found that flavor chemicals transfer very efficiently into EC aerosols^33^, and that refill fluid toxicity accurately predicts aerosol toxicity in about 74% of the cases^34^. These studies further showed that the solvents, particularly glycerol, increased toxicity when aerosols were produced in a tank style EC (iClear 16D dual coil clearomizer with Innokin battery) at higher power and that flavor chemicals produce potentially toxic reaction products when heated to create aerosols^34^. Thus, in aerosols, dominant flavor chemicals may combine with pyrolysis products from both the flavor chemicals and solvents to increase cytotoxicity beyond what was shown in the current study.

All 20 products would be predicted to be cytotoxic at 100% strength based on the concentrations of flavor chemicals in these products (Fig. 9), and this would be relevant to dermal exposure, in which fluids are not diluted. Even “Bird Brains”, which had low levels of flavor chemicals, had sufficient maltol (0.032 mg/ml) to be cytotoxic at full strength. In fact, maltol was used in 18 of 20 products at concentrations that would be predicted to be cytotoxic in the undiluted refill fluid. However, the concentrations of the flavor chemicals reaching the lungs and other organs have not yet been directly measured in humans and are probably quite variable given the large differences reported in EC user puffing topography^35^. Table 2 summarizes the concentrations of flavor chemicals in refill fluids (maximum observed concentration is given for each chemical), the amount of each chemical a user would be exposed to if they inhaled 3.4 ml/day (average consumption reported previously) for 2 days^36^, and how the *in vitro* IC_70_ and IC_50_ compare to the estimated consumption. As can be seen in Table 2, the intake of flavor chemicals is high enough to be a concern based on the *in vitro* cytotoxicity data.

**Table 2:** Extrapolated Daily Consumption of Flavor Chemicals by EC Users.

Flavor Chemicals	Concentration in Refill Fluids (mg/ml)^a^	Projected Human Consumption (mg/ 2 day)^b^	mNSC		BEAS-2B	
			IC_70_^c^ (mg/ml)	IC_50_^d^ (mg/ml)	IC_70_ (mg/ml)	IC_50_ (mg/ml)
Ethyl Maltol	6.5	44.2	0.01	0.03	0.03	0.06
Furaneol	1.2	8.2	0.02	0.03	0.03	0.11
Maltol	1.8	12.2	0.02	0.04	0.03	0.04
Ethyl Vanillin	6.6	44.8	0.13	0.24	0.11	0.25
Vanillin	6.8	46.2	0.15	0.38	0.15	0.32
Benzyl Alcohol	1.9	13	0.20	0.31	0.72	1.87
Ethyl Butanoate	2.7	18.4	0.37	0.72	>2.7	>2.7
Triacetin	5.8	39.4	0.84	1.24	4.30	5.20
Acetoin	1.7	11.6	>1.7	>1.7	>1.7	>1.7
Ethyl Acetate	1.6	10.8	>1.6	>1.6	>1.6	>1.6

^a^mg/ml = highest concentration of flavor chemicals in the two most cytotoxic refill fluids.

^b^mg/2 day = exposures based on consumption of 3.4 mL of EC refill fluid over a 2-day period.

^d^IC_70_ = values read directly off the dose response curves.

^c^IC_50_ = values obtained after non-linear fit using log (inhibitor) vs. normalized response - variable slope model.

Little is known about the specific effects of inhaled flavor chemicals on cells of the respiratory system or disease progression of EC users. Most toxicological work with flavor chemicals has been done on ingestion, and those studies that have evaluated inhalation toxicity have generally used animal models, not humans. Of the eight chemicals we tested in the MTT assay, only two have been examined in inhalation studies with rats, in which fatality was the endpoint^17^. For vanillin, inhalation of 41 mg/kg/2 hours was fatal in rats, whereas a much higher dose (3300 mg/kg) produced fatality by ingestion^17^, demonstrating that for this example, the FEMA GRAS designation would not be valid for inhalation. The best characterized of the flavor chemicals with respect to human effect is diacetyl, which as mentioned above, has been linked to bronchiolitis obliterans in humans^12,13^. Diacetyl was present in EC refill fluids at relatively low concentrations, which nevertheless are high enough to be a concern. Many of the flavor chemicals in EC products are aldehydes, which are highly reactive and usually cause irritation and inflammation of the respiratory epithelium^17^. Cinnamaldehyde is particularly noteworthy as it is highly toxic *in vitro* at low concentrations^10,33^. EC users have apparently experienced adverse health effects with its use as some bloggers have recommended avoiding products with cinnamon flavors, which are also known to rapidly etch plastic tanks, indicative of its reactivity.

The MTT assay is frequently used to evaluate cytotoxicity^37^ and to provide information on the health of mitochondria. Because many lung diseases are characterized by defects in mitochondrial function^38^, the MTT also provides insight into possible diseases that could be linked to flavor chemicals. For example, oxidative phosphorylation is often impaired in COPD, asthma, and lung cancer^38^. While there have been relatively few case reports related to EC use, those that do exist often include lung disease and most of these involve inflammation^5^ Mitochondria play a key role in lung homeostasis and proper functioning of lung immune cells^38^, and one study has linked impairment of innate immune cell response to cinnamaldehyde^11^, a flavor often used in EC products^10^. The limited data currently available demonstrate that flavor chemicals do affect mitochondrial function *in vitro* and establish the need for a better understanding of this finding on disease progression in EC users.

Our study was done using submerged cultures which are particularly valuable for screening purposes and for identification of those flavor chemicals that would be most interesting to study further in air-liquid interface systems, which we are currently doing, and in human inhalation studies. It will also be important in future work to determine if reaction products form heated flavor chemicals that could affect the cytotoxicity of aerosols and if flavor chemicals produce adverse effects *in vivo*.

## Conclusions

“Berries/Fruits/Citrus” flavored refill fluids were the most popular in three independent methods of analysis. The 20 popular refill fluids contained 22 to 47 different flavor chemicals with their total concentrations ranging from 0.63 to 27.9 mg/ml. Eighteen flavor chemicals were present in at least one refill fluid at a concentration ≥1 mg/ml. 80% of the 20 popular products were cytotoxic in the MTT assay. The four most cytotoxic refill fluids contained various combinations of the six chemicals (ethyl maltol, furaneol, maltol, ethyl vanillin, benzyl alcohol, and vanillin) that were cytotoxic as authentic standards. Most of these chemicals were present in the cytotoxic refill fluids at concentrations >1 mg/ml. Maltol and ethyl maltol, which were highly toxic, were present in 19 and 13 of the 20 refill fluids, respectively. The cytotoxicity of refill fluids was directly correlated with ethyl maltol concentrations in the fluids. These data raise concerns about the safety of popular EC refill fluids as those tested all contained concentrations of flavor chemicals that would be cytotoxic at the concentration in the undiluted fluids (Fig. 9). Although flavor chemicals have been used for many years in foods, their introduction into products that are heated and inhaled presents new potential health concerns. Our data may facilitate establishing concentration limits of the dominant flavor chemicals used in EC refill fluids and requirements for labeling the flavor chemicals included in each product.

## Materials and Methods

### Design, Recruitment, and Analysis for the Online Survey

Our study was approved by the Institutional Review Board at UC Riverside. Informed consent was not required for the survey which did not involve direct interaction with human subjects. An online survey was created with Survey Monkey using filter logic. The survey contained questions pertaining to EC users: (1) preference for refill fluid flavors and (2) conventional smoking and EC use history.

To obtain a broad cross section of EC users, survey participants were recruited from: (1) UC Riverside between May 2015 to August 2015 via email; and (2) various online health forums (WebMD, DailyStrength, eHealthForum, and Student Doctor Network), and (3) sites with special interest groups related to EC use or survey volunteering (Craigslist, Reddit). The resulting EC user data were analyzed to determine user demographics, EC usage history, and flavor preferences.

### Identification of EC flavor preferences in Southern California shops and online stores

Each product was assigned an inventory number and stored at 4 °C. For each refill fluid, 50 µl was diluted with 0.95 ml of isopropyl alcohol (Fisher Scientific, Fair Lawn, NJ) for an overall dilution ratio of 20 to 1. All diluted samples were shipped overnight on ice to Portland State University and analyzed on the day they were received using gas chromatography/mass spectrometry (GC/MS) to identify and quantify the flavor chemicals. Twenty µl of internal standard (2000 ng/µl of 1,2,3-trichlorobenzene) were added into each sample before GC/MS analysis. Using internal standard-based calibration procedures described elsewhere^39^, analyses were performed with an Agilent 5975 C GC/MS system (Santa Clara, CA). A Restek Rxi-624Sil MS column (Bellefonte, PA) was used (30 m long, 0.25 mm id, and 1.4 µm film thickness). One µl of each sample was injected into the GC with a 10:1 split. The injector temperature was 235 °C. The GC temperature program for all analyses was: 40 °C hold for 2 min; 10 °C/min to 100 °C; then 12 °C/min to 280 °C; hold for 8 min at 280 °C; then 10 °C/min to 230 °C. The MS was operated in electron impact ionization mode at 70 eV in positive ion mode. The ion source temperature was 220 °C. The scan range was 34 to 400 amu. Each target analyte (178 total) was quantitated using authentic standards (pure chemicals) and an internal standard (1,2,3-trichlorobenzene) normalized multipoint calibration. All reported concentration values were based on the 20:1 dilution sample except for overloaded peaks at 20:1 dilution, in which case quantitation was based on a 400:1 dilution sample.

### Cell Culture

Concentration-response cytotoxicity experiments were performed using mouse neural stem cells (mNSC) and human bronchial epithelial cells (BEAS-2B). The mNSC and BEAS-2B measure cytotoxicity in a neurological and respiratory model. In addition, mNSC provide information on a stem cell population as well as data that can be compared to our earlier studies^8^, and they are robust in moderate throughput assays. Cytotoxicity was measured using the MTT assay (MTT = 3-(4,5-dimethylthiazol-2-yl)-2,5-diphenyltetrazolium bromide), which measures the reduction of a yellow tetrazolium bromide to a purple formazan. This assay is widely used to screen for toxicity.

The mNSC were cultured in Dulbecco’s Eagle’s Medium (DMEM) (Lonza Walkersville, MD) supplemented with 10% fetal bovine serum, 5% horse serum, and 1% each penicillin-streptomycin (GIBCO, Invitrogen, Carlsbad, CA) and sodium pyruvate. Nunc T-25 tissue culture flasks (Fisher Scientific, Tustin, CA) were used to culture cells, and medium was replaced every 48 hours. At 80% confluency, cells were harvested using Dulbecco’s phosphate buffered saline (DPBS) for washing and incubated with 0.05% trypsin EDTA/DPBS (GIBCO, Invitrogen, Carlsbad, CA) for 2 mins at 37 °C to allow detachment from the culture flask. For the MTT assay, plating was done at 1,500 cells/well in 96 well plates.

Human BEAS-2B cells were purchased from the American Type Culture Collection (ATCC, USA). The cell line was cultured in basal bronchial epithelial cell basal medium (BEBM) (Lonza, Walkersville, MD) supplemented with 2 ml of bovine pituitary extract and 0.5 ml of: insulin, hydrocortisone, retinoic acid, transferrin, triiodothyronine, epinephrine, and human recombinant epidermal growth factor (Lonza, Walkersville, MD). Nunc T-25 tissue culture flasks were coated overnight with BEBM, collagen, bovine serum albumin (BSA) and fibronectin prior to culturing and passaging cells. At 80% flask confluency, cells were harvested using DPBS for washing and incubated with 1.5 ml of 0.25% trypsin EDTA/DPBS and poly-vinyl-pyrrolidone for 3–4 mins at 37°C to allow detachment. Cells were cultured in T-25 flasks at 75,000 cells/flask, and medium was replaced the next day and then every other day. Plating for the MTT assay was done at 3,500 cells/well in pre-coated 96-well plates.

### Cytotoxicity of EC refill fluids

The cytotoxicities of the 20 EC refill fluids were evaluated in 96-well plates using the MTT assay^37,40^. Serial dilutions (0.001–1%) of refill fluids were made in culture medium and arranged in 96-well plates with a negative control (culture medium only) adjacent to the high and low concentration to check for a vapor effect. The high concentration of 1% was chosen as preliminary experiments showed that it did not produce a vapor effect in this study. mNSC were added to non-coated 96-well plates at 1,500 cells/well, allowed to attach for 24 hours, then treated for 48 hours with serial dilutions of refill fluids. After treatment, 20 µl of MTT (Sigma-Aldrich, St Louis, MO) dissolved in 5 mg/ml of DPBS (Fisher Scientific, Chino, CA) were added to each well and incubated for 2 hrs at 37 °C. Solutions were removed and 100 µl of dimethyl sulfoxide (DMSO) (Fisher Scientific, Chino, CA) were added to each well and gently mixed by pipetting until homogenous. Absorbance of control and treated wells was read against a DMSO blank at 570 nm using an Epoch micro-plate reader (BioTek, Winooski, VT).

### Cytotoxicity of authentic standards of flavor chemicals

A heat map of the flavor chemicals found in the two most cytotoxic refill fluids and their clones was examined to identify potentially toxic flavor chemicals that were present in high concentrations. Authentic standards of each chemical (ethyl maltol, maltol, ethyl vanillin, vanillin, benzyl alcohol, and furaneol) (Sigma-Aldrich, St. Louis, MO) diluted in culture medium were tested individually using the MTT assay with mNSC and BEAS-2B cells. Toxicity assays were performed over a concentration range that included the concentration of each chemical found in the refill fluids.

### Data analysis

The MTT assay was performed in three independent experiments for each refill fluid and authentic standard chemical. Data were normalized by setting treatment wells as percentages of the negative control (100%). Graphs were plotted using GraphPad Software (GraphPad, San Diego, CA.), and significance was obtained using a one-way analysis of variance (ANOVA) followed by Dunnett’s post hoc test in which treated groups were compared to the lowest concentration. The ANOVAs were used to determine which concentrations of refill fluid or authentic standard produced a significant effect in the MTT assay. GraphPad Prism software was also used to compute IC_50s_ with the log inhibition vs. normalized response-variable slope with the top and bottom constraints set to 100% and 0%, respectively.

## Supplementary information

Supplemental Figures and Tables
