# Supplementary material for: Identification of Cytotoxic Flavor Chemicals in Top-Selling Electronic Cigarette Refill Fluids
Source: Sci Rep. 2019 Feb 26;9:2782. doi: 10.1038/s41598-019-38978-w (PMC6391497; doi:10.1038/s41598-019-38978-w)
Supplement: Supplementary file 1 — Supplemental Figures and Tables [file 41598_2019_38978_MOESM1_ESM.pdf]

Supplemental Material

Identification of cytotoxic flavor chemicals in top selling electronic cigarette refill fluids

My Hua BA, BS, Esther E. Omaiye, BS, MS, Wentai Luo, Ph.D, Kevin J. McWhirter BS, James F. Pankow, Ph.D, and Prue Talbot, Ph.D

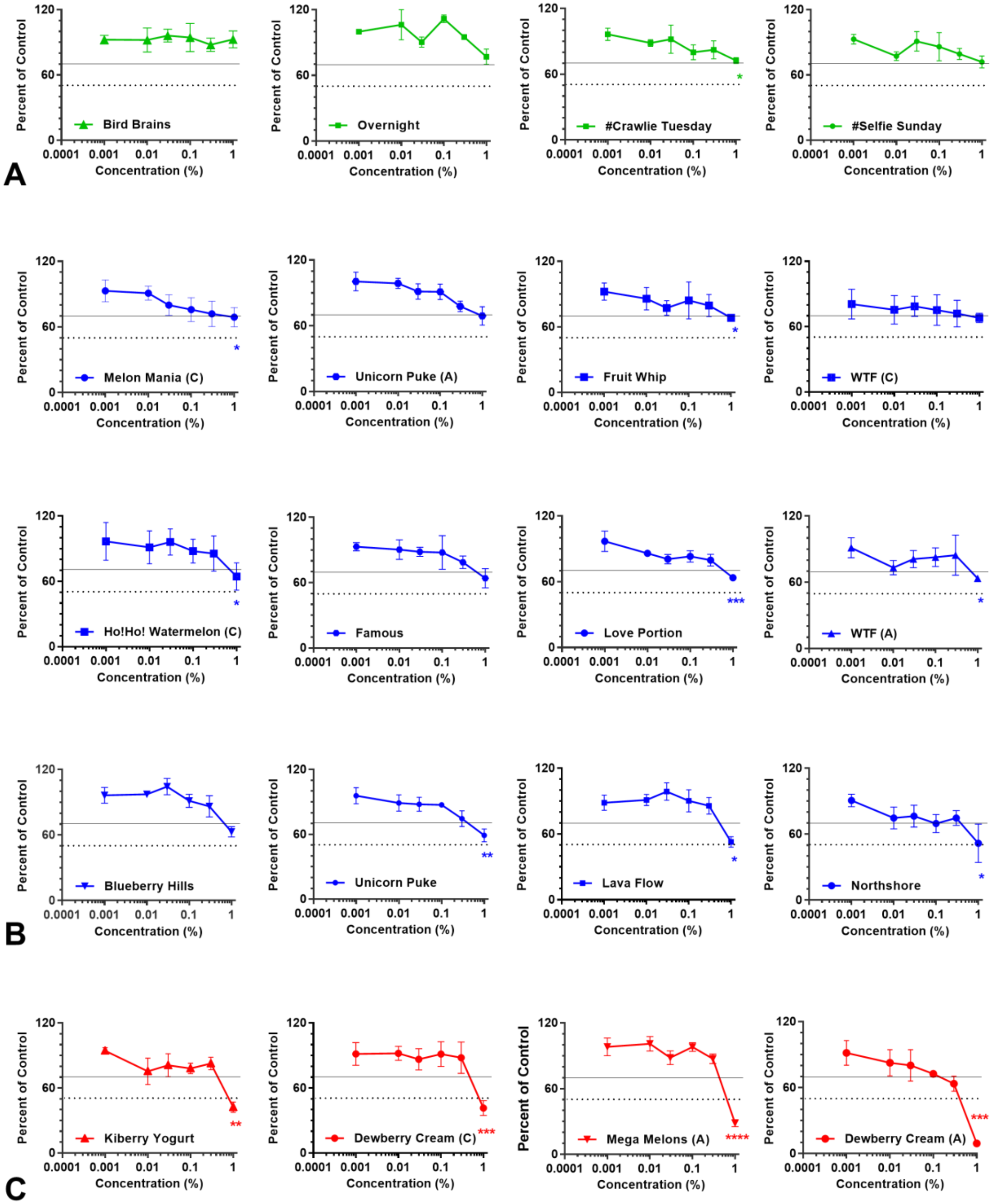

Supplemental Figure 1. Dose-response curves for 20 popular refill fluids. (A) Non-cytotoxic fluids. (B) Fluids reaching an IC_{70}. (C) Fluids reaching an IC_{50}. Horizontal bars are at the IC_{70} and IC_{50}. Each graph is the mean ± the std error of the mean for three independent experiments. * = $p<0.05$, ** = $p<0.01$, *** = $p< 0.001$, **** = $p< 0.0001$.

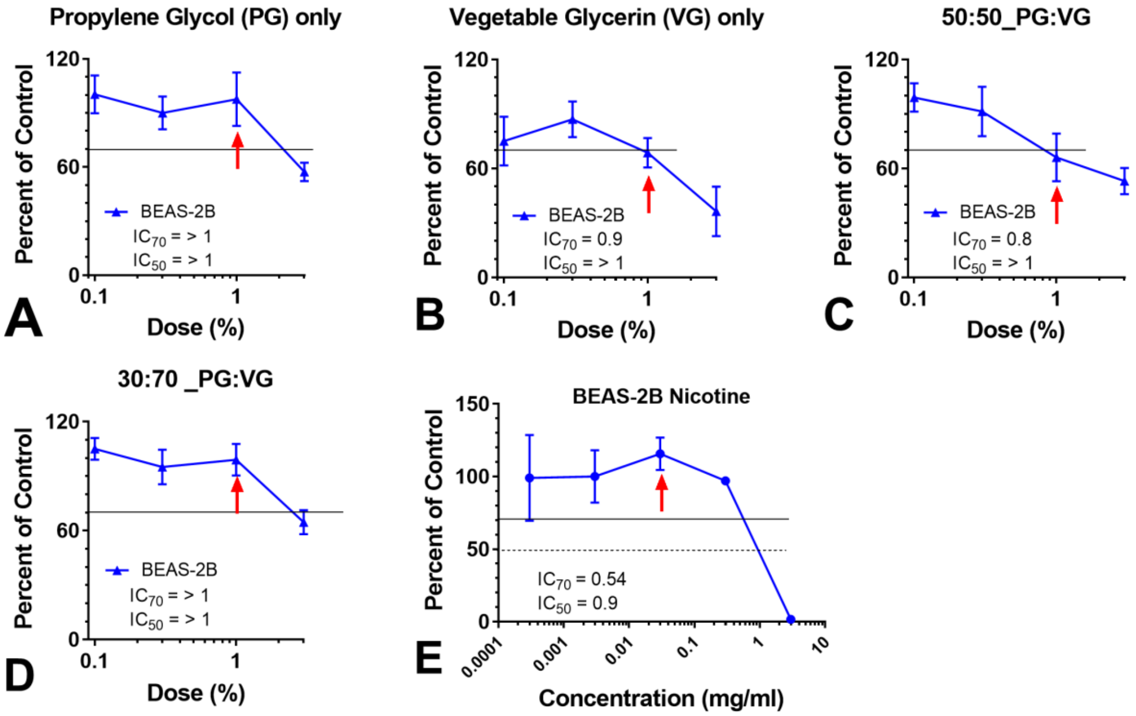

Supplemental Figure 2. Dose-response curves for propylene glycol, glycerol and nicotine. (A) Propylene glycol (PG). (B) Glycerol (C) 50:50 PG: VG (D) 30:70 PG: VG (E) Nicotine. The red arrows indicate the highest concentrations of solvents and nicotine that were used in the current study. Horizontal bars are at the IC_{70} and IC_{50}. Each graph is the mean ± the std error of the mean for three independent experiments.

Supplemental Table 1. EC User Demographics

**General Demographics**		**All EC Users (N=835)**
Age at survey	<14	0 (0%)
	14-17	9 (1.08%)
	18-22	413 (49.46%)
	23-27	211 (25.27%)
	28-32	84 (10.06%)
	33-42	75 (8.98%)
	43-50+	43 (5.16%)
Gender	Male	599 (71.74%)
	Female	231 (27.66%)
	Other	5 (0.60%)
Race/Ethnicity	Asian	237 (28.38%)
	Black or African American	26 (3.11%)
	Hispanic/Latino	158 (18.92%)
	Indian	16 (1.92%)
	Middle Eastern	39 (4.67%)
	Native American or Alaska Native	80 (9.58%)
	Native Hawaiian or Pacific Islander	16 (1.92%)
	White/Caucasian	361 (43.23%)
	Other	13 (1.56%)
Highest Education	No high school	2(0.24%)
	Some high school	12 (1.44%)
	High school graduate or GED equivalent	122 (14.61%)
	Certification work	78 (9.34%)
	Some college	325 (38.92%)
	2-year college degree	74 (8.86%)
	4-year college degree	151 (18.08%)
	Some post-graduate education	22 (2.63%)
	Graduate or professional school	49 (5.87%)
**EC Use History**		
Age First Vaped	<14	3 (0.36%)
	14-17	218 (26.11%)
	18-22	413 (49.46%)
	23-27	211 (25.27%)
	28-32	84 (10.06%)
	33-42	75 (8.98%)
	43-50+	43 (5.16%)
Contributed to Vaping*	People in my family vape	74 (8.86%)
	Friends of mine vape	454 (54.37%)
	I saw people in the media vaping	128 (15.33%)
	I wanted to stop smoking conventional cigarettes	365 (43.71%)
	Vaping is a stress reliever	262 (31.38%)
	Vaping lowers my appetite	75 (8.98%)
	Vaping is safer than conventional cigarette smoking	462 (55.33%)
	I live or work near a vape shop	118 (14.13%)
	Other	55 (6.59%)
Length of EC Usage	<1 month	37 (4.46%)
	1-6 months	200 (24.10%)

	7-11 months	89 (10.72%)
	1 year	226 (27.23%)
	2 year	185 (22.29%)
	3-4 year	74 (8.92%)
	5+ year	19 (2.28%)
EC Frequency Use	Regularly, at least once a day	470 (56.63%)
	Occasionally	153 (18.43%)
	Rarely	139 (16.75%)
	Socially	68 (8.19%)
Hao often have you vaped in the past 30 days?		
	None	39 (4.70%)
	Less than once a week	128 (15.42%)
	A few times a week (less than five times a week)	212 (25.54%)
	Daily or almost daily	451 (54.34%)
Daily Vaping	Yes	677 (81.57%)
	No	153 (18.43%)
EC Use Daily	<10 minute intervals per day	131 (19.47%)
	10-30 minute sessions a day	128 (19.02%)
	31-59 minutes a day	163 (24.22%)
	1-3 hours a day	146 (21.69%)
	4-6 hours a day	56 (8.02%)
	7+ hours a day	51 (7.58%)

Supplemental Table 2. Flavor Profiles for Popular Brands

Brand Names [StoreID]	Generic Flavor Profile and General Category
Dewberry Cream [3]	Honeydew; mixed berries; cream (Berries/Fruits/Citrus; Buttery/Creamy/Caramel/Vanilla)
Blueberry Hills [4]	Blueberry pop tart (Berries/Fruits/Citrus; Bakery/Dessert)
Dewberry Cream [1c]	Honeydew; mixed berries; cream (Berries/Fruits/Citrus; Buttery/Creamy/Caramel/Vanilla)
Rainbow Sherbet [1c]	Rainbow sherbet ice cream (Berries/Fruits/Citrus; Buttery/Creamy/Caramel/Vanilla)
Kiberry Yogurt [3]	Kiwi; yogurt (Berries/Fruits/Citrus; Buttery/Creamy/Caramel/Vanilla)
WTF [1c]	Strawberry; sourbelt candy (Berries/Fruits/Citrus; Candy)
Mega Melons [3]	Melon; cantaloupe; papaya; mango (Berries/Fruits/Citrus)
Lava Flow [3]	Strawberry; pineapple; coconut (Berries/Fruits/Citrus)
Unicorn Puke [4]	Rainbow sherbert ice cream (Berries/Fruits/Citrus; Buttery/Creamy/Caramel/Vanilla)
#Crawlie Tuesday [2]	Gummy worm candy (Berries/Fruits/Citrus; Candy)
Famous [4]	Strawberry; watermelon; pineapple; citrus (Berries/Fruits/Citrus)
Fruit Whip [3]	Apples; pear; berries; cream (Berries/Fruits/Citrus; Buttery/Creamy/Caramel/Vanilla)
North Shore [4]	Dragon fruit; guava; papaya (Berries/Fruits/Citrus)
Love Potion [2]	Apple; menthol (Berries/Fruits/Citrus; Menthol)
Ho!Ho! Watermelon [1]	Watermelon; assorted melons (Berries/Fruits/Citrus)
WTF [2]	Strawberry; sourbelt candy (Berries/Fruits/Citrus; Candy)
Melon Mania [1c]	Assorted melons (Berries/Fruits/Citrus)
#Selfie Sunday [2]	Apple (Berries/Fruits/Citrus)
Overnight [4]	Strawberry; watermelon; Jolly rancher candy (Berries/Fruits/Citrus; Candy)
Bird Brains [2]	Fruit loop cereal (Berries/Fruits/Citrus; Breakfast Cereal)

Supplemental Table 3. Chemical Flavor Compounds and their Descriptive Taste Profiles

**Flavor Chemical**	**Descriptive Taste Profile(s)**
Furfural	*Bready, brown, burnt nuance, caramellic nuance, nutty, woody*
Allyl hexanoate	*Fresh, fruity, juicy nuance, pineapple, sweet*
Isopulegol	*Cool, mint*
1-Pentanol	*Bready, cereal, fruity undernote, fermented, intense fusel*
Furfuryl alcohol	*Burnt, creamy, caramellic, milky, powdery*
Coumarin	*aromatic nuance, bitter, burning*
Guaiacol (2-methoxyphenol)	*Bacon, phenolic, medicinal, savory, smoky, woody*
1-Hexanol	*Apple skin, fruity, green nuance, oily*
Acetophenone	*Almond, cherry pit-like, coumarinic nuances, fruity nuances, powdery*
Methyl salicylate	*Aromatic nuance, balsamic nuance, root beer, salicylate, sweet*
Ethyl maltol	*Burnt, cotton sugar, candy-like, jammy notes, strawberry notes, sweet*
Isopentyl alcohol	*Banana, cognac, etherial, fermented, fruity, fusel*
Benzaldehyde	*Sweet, oily, almond, cherry, nutty and woody*
Raspberry ketone	*Berry, blueberry, cotton candy nuances, fruity, jammy notes, raspberry, seedy nuances*
Maltol	*Berry notes, caramelic, cotton candy, fruity notes, jammy notes, sweet*
Hydrocoumarin	*Coconut, creamy, milky, sweet, vanilla*
Benzyl alcohol	*balsamic nuance, chemical, fruity nuances*
2,3-Butanedione	*Buttery, creamy, milky, sweet*
Vanillin	*Creamy, milky, phenolic, spicy, sweet, vanilla*
Ethyl vanillin	*Caramellic, creamy, smooth, sweet, vanilla*
p-Tolualdehyde	*Cherry, deep phenolic, fruity*
Benzyl benzoate	*Balsamic, berry nuances, fruity, powdery nuance*
Phenethyl alcohol	*Bready, floral, rosy, sweet*
Furaneol	*Caramelic, cotton candy, sweet*
Eugenol	*Phenolic nuances, clove, spicy, sweet, woody nuances*
α-Pinene	*Cedarwood, pine, sharp*
β-Pinene	*Camphoraceous, fresh, minty, piney, spicy, terpy, woody*
p-Cymene	*Citrus notes, green pepper, oregano, rancid, spice nuance, terpy, woody*
Carvone	*Caraway, minty, spicy*
Cinnamaldehyde	*Cinnamon, spicy*
Hexyl acetate	*Apple, banana peel, fresh, fruity, green, pear*
p-Anisaldehyde	*Almond, anise, mint*
Eucalyptol	*Eucalyptus*
Isosafroeugenol	*Creamy, anisic, sweet, vanilla-like*
Amyl acetate	*Apple, banana, ethereal, fruity, pear*
Isobutyl acetate	*Cereal, bready, fermented, fruity undertones, fusel*
Benzyl butyrate	*Apricot, fruity, pear, pineapple, sweet, tropical*
Cinnamyl alcohol	*Fermented, yeasty nuance, floral, green, honey, spicy*
Ethyl 2-methylbutanoate	*Berry, cherry notes, fruity, fresh, grape, pineapple, mango*
Strawberry Glycidate_B	*Berry, floral, fruity, strawberry, sweet, tutti frutti*
Hemineurine	*Brothy, meaty, metallic, nutty, roasted*
Benzyl acetate	*Balsamic undernotes, fruity, jasmine floral undernotes, sweet*
Methyl cinnamate	*Balsamic, cinnamyl, fruity, strawberry*
Piperonal	*Benzaldehyde, cherry, spicy, vanilla*
Linalool	*Aldehydic, citrus, floral, lemon, orange, waxy, woody*
Methyl anthranilate	*Berry nuances, concord grape, fruity, musty nuances, sweet*

β-Damascone	*Floral, green, herbal, minty, woody*
2,3-Pentanedione	*Buttery, caramellic, marshmellow nuance, molasses nuance, toasted*
Benzaldehyde PG acetal	*Floral*
Acetylpyrazine	*Bready, corn chip nuance, nutty, popcorn nuance, roasted, yeasty nuance*
Menthol	*Cool, mint*
(E)-2-Hexen-1-ol	*Fatty, fresh, fruity nuance, green nuance, juicy nuance*
Ethyl propanoate	*Apple nuance, bubble gum, etherial, fruity, grape nuance, sweet, winey*
6-Methyl-5-heptene-2-one	*Apple, banana, green bean, green, mistry, vegetative*
trans-Geraniol	*Floral, fruity, peach-like nuance, rosy, waxy*
γ-Terpinene	*Citrus, green, lime, oily, terpy, tropical fruity nuance*
2-Methylbutyl acetate	*Banana, ripe, fruity, juicy nuance, sweet*
Ethyl cinnamate	*Balsamic, berry, fruity, green, powdery, punch, spice, sweet*
α-Terpineol	*Anise, fresh, mint, oil*
δ-Decalactone	*Buttery, coconut, creamy, fatty, fruity nuance, milky, nutty*
γ-Octalactone	*Apricot, coconut, courmarin, creamy, fruity, lactonic, peach, toasted*
α-Ionone	*Berry, floral, fruity, raspberry, strawberry, violet*
(3Z)-3-Hexen-1-ol	*Fresh, fruity nuance, green nuance, raw nuance*
Citral	*Citrus, green, herbal, juicy, lemon peel, lime, woody*
Strawberry Glycidate_A	*Berry, floral, fruity, strawberry, sweet, tutti frutti*
Fenchol	*Fresh, pine*
Acetoin	*Buttery, creamy, dairy, milky, oily, sweet*
Isoamyl butyrate	*Berry notes, estry nuance, fruity, green apple, melon notes, sweet, waxy*
Isopentyl phenylacetate	*Chocolate nuances, dried fruit notes, honey*
γ-Decalactone	*Apricot, creamy, fatty nuance, fruity, peachy syrupy nuance*
Hexyl 2-methylbutyrate	*Apple, banana, fresh, fleshy nuance, green, unripe, fruity, waxy*
Ethyl isovalerate	*Apple, fruity, green, metallic, pineapple, spice, sweet*
Nerol acetate	*Floral, fruity, pear, rosy, soapy, tropical*
δ-Dodecalactone	*Buttery, creamy, dairy, fatty, nutty, peach,*
Ethyl hexanoate	*Banana, estry nuance, fruity, green, pineapple, sweet, waxy*
Butyl butyrolactate	*Cheesy nuances, creamy, dairy, fatty, milky, waxy*
Limonene	*Camphoraceous, citrus, herbal, terpene*
Benzyl cinnamate	*Balsamic, floral, fruity, spicy*
Ethyl Acetate	*Cherry, nuance, etherial, fruity, grape, nuance, sweet*
Geraniol Acetate	*Citrus nuances, floral, green, oily, rum nuances, soapy, waxy, wine nuances*
γ-Nonalactone	*Coconut, creamy, milky notes, waxy*
(E)-β-Ionone	*Berry, floral, fruity, powdery nuances, woody*
Ethyl lactate	*Creamy, caramellic nuance, fruity, sweet, pineapple*
Butyl butyrate	*Fatty, fresh, fruity, sweet*
Linalyl acetate	*Citrus, floral, green, herbal nuances, spicy nuances, terpy, waxy*
Ethyl butanoate	*Apple, etherial, fresh, fruity, sweet, tutti frutti*
Isoamyl isovalerate	*Apple, fruity nuance, green*
Isoamyl acetate	*Banana, estry nuance, fruity, green nuance, ripe nuance, sweet*
δ-Undecalactone	*Coconut, creamy, fatty, macadamia, nutty, peach, vanilla*
Ethyl heptanoate	*Banana, fruity, oily nuance, pineapple, spicy nuance, strawberry*
Heliotropine PG acetal	*Floral, fruity*
Hydroxyacetone	*Burnt, sweet, slightly green nuance*
Corylone	*Bready, caramelic, maple, nutty nuances, sweet*
Triacetin	*Creamy, oily nuance*
1,4-Cineole	*Camphoraceous, cooling, green, herbal, menthol, terpy*

Benzyl propionate	*Apple, banana, floral nuance, fruity, sweet, tutti frutti*
(Z)-3-Hexen-1-ol, acetate	*Apple, green, pear, tropical fruity nuance*
Amyl isovalerate	*Apple, fresh, fruity*
cis-Limonene oxide	*Cool, mint*

Supplemental Table 4. List of Chemicals Not Included in Figure 2

(3Z)-3-Hexenyl formate
1,2-Dihydrolinalool
2,3,5,6-Tetramethylpyrazine
2,3,5-Trimethylpyrazine
2,3-Dimethylpyrazine
2,3-Hexanedione
2,5-Dimethylpyrazine
2-Acetylpyrrole
2-Ethyl-3-methylpyrazine
2-Hydroxy-3,5,5-trimethyl-cyclohex-2-one
2-Methoxy-3-methylpyrazine
2-Methylbenzofuran
2-Nonanone
3'-Methylacetophenone
4-Methylbenzyl alcohol
4-Terpineol
α-Caryophyllene (α-Humulene)
α-Damascone
β-Caryophyllene
β-Citronellal
β-Myrcene
γ-Heptalactone
γ-Pentalactone
Acetyleugenol
Amyl butyrate
Aromadendrene
Benzeneacetaldehyde
Benzeneacetic acid, ethyl ester
Benzoin ethyl ether
Benzophenone
Benzyl benzeneacetate
Benzyl dimethylcarbinyl butyrate
Benzyl ether
Benzylacetaldehyde
Butyl acetate
Caffeine
Cinnamyl acetate
cis-Linalool oxide
Citronellyl propionate
Coumarin, 6-methyl
D-Neomenthol
Dimethyl butanedioate
Estragole (4-allylanisole)
Ethyl 3-hydroxybutyrate
Ethyl anthranilate
Ethyl benzoate
Ethyl benzoylformate
Ethyl decanoate
Ethyl isobutyrate
Ethyl laurate
Ethyl nonanoate
Ethyl octanoate
Ethyl salicylate
Eugenol methyl ether
Gingerone
Hexanal
Hexyl hexanoate
Hexyl octanoate
Isoamyl propionate
Isoeugenol methyl ether
Linalyl propionate
Menthyl acetate
Methyl 2-methylbutyrate
Methyl 2-octynoate
Methyl N-methylanthranilate
Methyl phenylacetate
Myristicin
o-Methoxycinnamaldehyde
p-Menthanone
Pentyl propanoate
Piperitone
Pulegone
Raspberry ketone methyl ether
Styralyl acetate
Syringol
Thymol
trans-D-Limonene oxide
trans-Linalool oxide

Supplemental Table 5. Flavor Chemicals Found in Only One Product

Chemical Category/Classification	Chemical Flavor Ingredient
**Toxic**	*Furfural*
**Harmful**	*Isopulegol*<br>*Coumarin*<br>*Acetophenone*<br>*Methyl salicylate*<br>*p-Tolualdehyde*<br>*Eugenol*
**Irritant**	*Carvone*<br>*Eucalyptol*<br>*Cinnamyl alcohol*<br>*Menthol*<br>*Fenchol*<br>*Isopentyl phenylacetate*<br>*Benzyl cinnamate*<br>*Butyl butyrate*<br>*Linalyl acetate*<br>*Ethyl heptanoate*
**No data**	*1,4-Cineole*<br>*Benzyl propionate*<br>*cis-Limonene oxide*
